# Episodic Vertigo: A Narrative Review Based on a Single-Center Clinical Experience

**DOI:** 10.3390/audiolres13060074

**Published:** 2023-11-01

**Authors:** Augusto Pietro Casani, Mauro Gufoni, Nicola Ducci

**Affiliations:** ENT Section, Medical, Molecular and Critical Area, Department of Surgical Pathology, Pisa University Hospital, 56122 Pisa, Italy; mgufoni@gmail.com (M.G.); n.ducci@yahoo.it (N.D.)

**Keywords:** episodic vertigo, imbalance, acute vertigo, vestibular migraine, Menière’s disease, otoneurologic examination, vestibular tests, benign paroxysmal positional vertigo

## Abstract

(1) Background: Usually, the majority of patients suffering from vertigo and dizziness can be identified in four major categories: acute spontaneous vertigo, episodic (recurrent) vertigo, recurrent positional vertigo, and chronic imbalance. Our purpose is to retrospectively evaluate the main causes of episodic vertigo and to find indications for a reliable clinical suspicion useful for a definitive diagnosis, comparing patients affected by different presenting symptomatology (acute vertigo, recurrent episodic vertigo, and imbalance). (2) Methods: we retrospectively evaluated the clinical records in a population of 249 consecutive patients observed for vertigo in our tertiary referral center in the period 1 January 2019–31 January 2020. On the basis of the reported clinical history, patients were divided into three groups: patients with their first ever attack of vertigo, patients with recurrent vertigo and dizziness, and patients with chronic imbalance. (3) Results: On the basis of the results of the instrumental examination, we arbitrarily divided (for each type of symptoms) the patients in a group with a normal vestibular instrumental examination and a group of patients in which the clinical–instrumental evaluation showed some pathological results; a highly significant difference (*p*: 0.157) was found between recurrent and acute vertigo and between recurrent vertigo and imbalance. (4) Conclusions: Patients with recurrent vertigo more frequently exhibit a negative otoneurological examination since they are often examined in the intercritical phase. A precise and in-depth research of the patient’s clinical history is the key to suspect or make a diagnosis together with the search for some instrumental or clinical hallmark, especially in cases where the clinical picture does not fully meet the international diagnostic criteria.

## 1. Introduction

Usually, the majority of patients suffering from vertigo and dizziness can be identified in four major categories [1]: 1. First-ever attack of acute isolated spontaneous vertigo; in these cases, there are two major diagnostic possibilities: acute unilateral vestibular loss (AUVL) or cerebellar infarction. In addition, benign paroxysmal positional vertigo (BPPV), especially when horizontal semicircular canal is involved, could manifest with an isolated episode of acute spontaneous vertigo [2,3]. 2. Recurrent positional vertigo, most often due to BPPV [4]; central positional vertigo is rarely due to a CNS involvement and usually needs to be considered in patients with persistence of positional vertigo or poor response to CRM [5]. 3. Recurrent spontaneous vertigo, commonly related to Menière’s Disease (MD) and vestibular migraine (VM), less frequently to vertebrobasilar transitory ischemic attacks (TIA) or vestibular paroxysmia [6]. 4. Imbalance: this symptom is commonly seen in patients with unilateral or bilateral vestibular loss or recurrent BPPV, and in a various central nervous system disorder as extrapyramidal syndromes, cerebellar ataxia, peripheral neuropathy, orthostatic hypotension, and side effects of some drugs [1]. In this category of patients are included patients with functional (psychogenic) dizziness, nowadays defined as affected by persistent postural–perceptual dizziness (PPPD) [7]. Between the above-mentioned group of symptoms, spontaneous and positional recurrent vertigo represents a pathological condition of relevant clinical importance given the large number of underlying causes. The recurrence of the vertigo attacks could lead to a disabling character with a high decline of the quality of life, inducing a feeling of insecurity and anxiety in the patient involving a series of additional pathological elements related to the involvement of the psycho-emotional sphere [6]. Frequently in patients with recurrent vertigo, both clinical (bedside) examination and testing results are often negative between attacks [1]. Diagnosis could be based on recognition of symptom clusters and temporal features by means of careful history taking. Furthermore, the physician cannot rely too much on technical investigations, whose results do not ever offer clear diagnostic indications. An important diagnostic clue could be related to the possibility to evaluate the patient suffering from acute vertigo in the acute phase of the disease, a condition not ever possible both for the short duration of the vertigo or for the impossibility for the patient to drive to the vertigo center.

Our purpose is to retrospectively evaluate the main causes of episodic vertigo and to find indications for a reliable clinical suspicion useful for a definitive diagnosis, comparing patients affected by different presenting symptomatology (acute vertigo, recurrent episodic vertigo, and imbalance).

## 2. Materials and Methods

We retrospectively evaluated the clinical records in a population of 249 consecutive patients observed for vertigo in our tertiary referral center in the period 1 January 2019–31 January 2020. On the basis of the reported clinical history, patients were divided into three groups:

(a) Patients with first ever attack of acute vertigo.
(b) Patients who reported recurring vertigo and dizziness (present or not at the time of observation) for at least three episodes, both spontaneous and provoked (for example, by movements of the head, change in body positions, physical efforts, etc.).
(c) Patients with chronic imbalance.

Patients underwent a thorough medical history, otoscopy, neurological evaluation (cerebellar tests and clinical evaluation of the cranial nerves), audiometry, evaluation of the spontaneous and positional nystagmus, head impulse test, skew deviation test (together with spontaneous and positional nystagmus as a part of HINTS paradigm), head shaking test and skull-vibration-induced nystagmus (SVIN) using infrared goggles. The instrumental examination consisted of performing video-HIT, caloric test, and cervical and ocular VEMPs. Patients with suspected central nervous system involvement underwent brain MRI. Patients with an acute prolonged attack of vertigo were observed for up to three–five days in order to evaluate the evolution of the symptoms. The statistical study was carried out with the GNU PSPP environments (Version 0.8.5) (Computer Software). Free Software Foundation. Boston, MA and JASP (JASP version 0.17.1 Computer software).

Ethical review and approval by the local Institutional Board (Comitato Etico Azienda Ospedaliero-Universitaria Pisana, Pisa, Italy) were waived for this study. Due to its retrospective nature, it was not set up as part of a research project. Furthermore, the study does not include new experimental diagnostic protocols, and the patients included in the study were diagnosed according to national guidelines. Written informed consent was obtained from all participants, and the study was conducted in accordance with the 1964 Declaration of Helsinki. 

## 3. Results

According to the clinical history, the 249 patients were divided as follows:

- 83 (53 females, 30 males, mean age ranging from 22 years to 85 years, mean 53.5 years) patients suffering from episodic vertigo and dizziness attacks.
- 89 (59 females, 30 males, mean age ranging from 29 years to 92 years, mean 60.5 years) with a first-ever attack of acute vertigo.
- 77 (48 females, 29 males, mean age ranging from 30 years to 99 years, mean 64.5 years) with imbalance.

**Figure 1:**
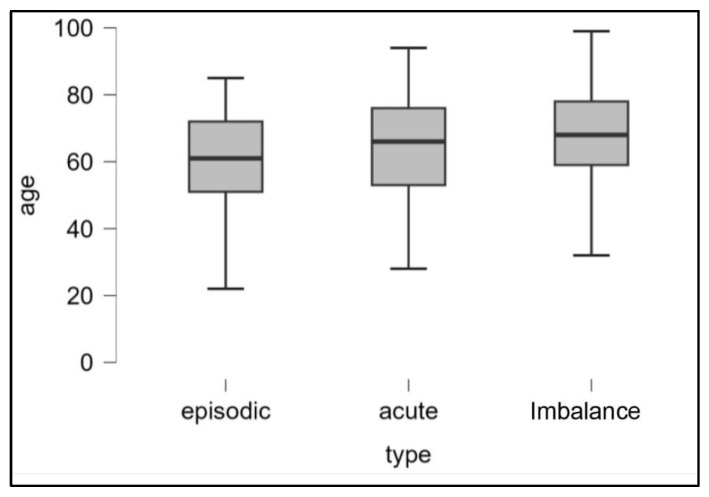
Representation (boxplot) of the age distributions of the three populations examined (episodic, acute crises, and imbalance).

We found no age difference between the ‘recurrent’ and ‘acute attack’ subgroups (*p*: 0.213) and between ‘acute attack’ and ‘imbalance’ (*p*: 0.189) while patients with episodic vertigo are younger than those with unsteadiness (*p* < 0.01) (Figure 1).

**Figure 2:**
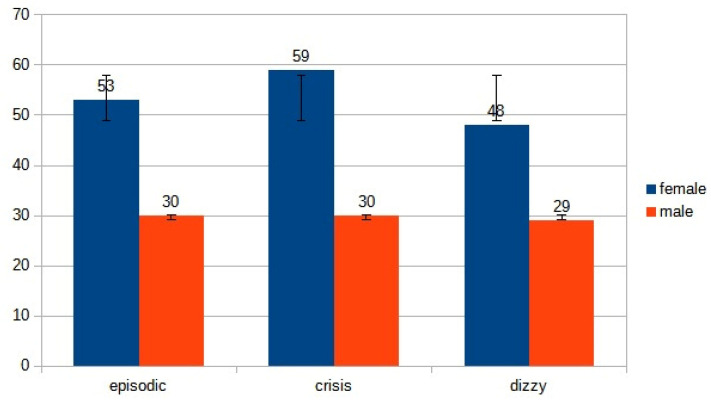
Distribution of gender in the three subgroups.

We found no gender difference between the three groups (recurrent versus acute attack *p*: 0.381; recurrent versus imbalance *p*: 0.367; acute attack versus imbalance *p*: 0.687), indicating a clear homogeneity of the population (Figure 2).

**Figure 3:**
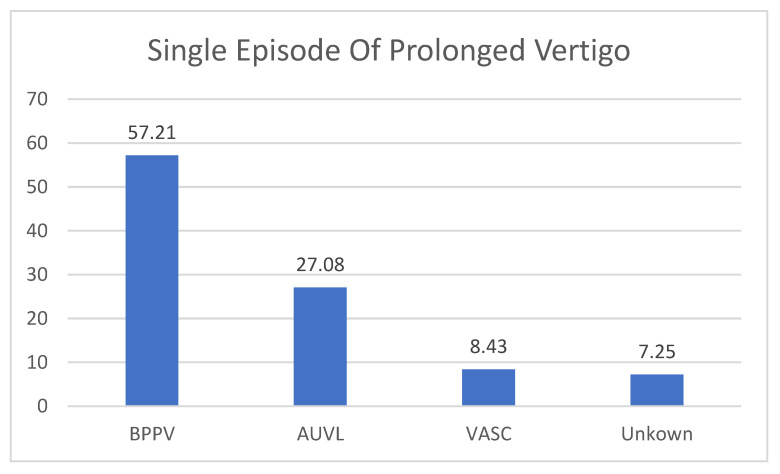
The diagnosis in patients with single episode of prolonged Vertigo. BPPV: Benign Paroxysmal Positional Vertigo (one single episode, diagnosis confirmed by positive response to liberatory maneuver); AUVL: Acute Unilateral Vestibular Loss; VASC: Vascular Vertigo (2 cases of cerebellar stroke, 5 cases of TIA; Unknown: no diagnosis was made. These are clinical diagnoses that do not necessarily coincide with the results of vestibular instrumental examination.

The 83 patients suffering from acute attacks of vertigo were diagnosed as affected by BPPV (47 patients, 57.21% of the cases) and AUVL in 23 patients (27.08% of the cases). A vascular origin of the vertigo was found in 7 (8.43%) patients. One patient suffered from VM, and another was classified as a first attack of MD. In 6 (7.25%) patients, the clinical and instrumental examination do not allow to drive a definite diagnosis (Figure 3). 

**Figure 4:**
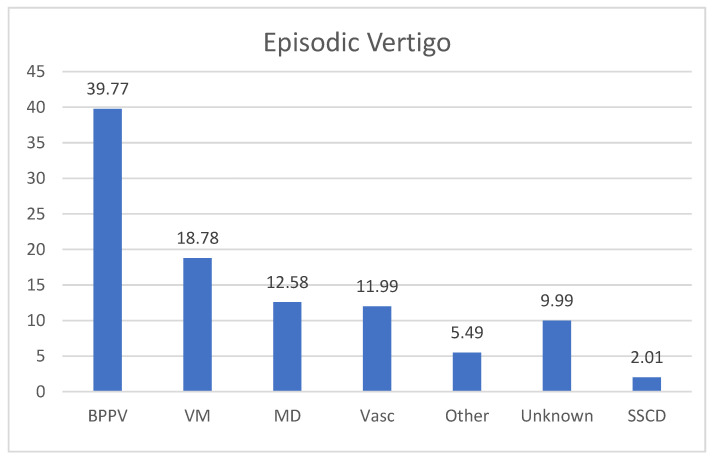
Final diagnosis in patients with episodic vertigo. BPPV: Benign Paroxysmal Positional Vertigo; VM: vestibular migraine; MD: Menière’s Disease; VASC: Vascular Vertigo (6 causes of TIA, 3 cases of SVD—Small Vessel Disease). SSCD: Superior Semicircular Canal Dehiscence Syndrome, 2 cases; Unknown: no diagnosis was made.

In the group of recurrent vertigo, the majority of the patients suffered from recurrent episodes of BPPV (35 patients, 39.77%); VM was diagnosed in 17 patients (18.78%) and 11 subjects (12.58%) were affected with MD. There were 10 (11.99%) patients diagnosed as affected with vascular vertigo. In 9 subjects (9.99%), no diagnosis was made (Figure 2). Other diagnoses (5 patients, 5.49%) included one case of autoimmune Systemic vasculitis, one case of multiple sclerosis, and one case the Arnold–Chiari malformation type 1, one case of perilymphatic fistula and one case of Orthostatic Hypotension (Figure 4). 

**Figure 5:**
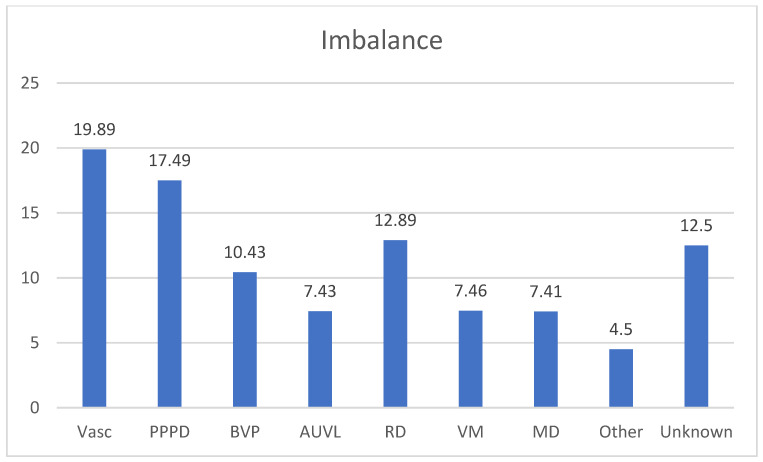
Percentage frequencies in the diagnosis of Unsteadiness. Vasc: Vascular causes, 15 patients of SVD (Small Vessel Disease) (19.89%); PPPD: Persistent Perceptual Postural Dizziness 13 patients (17.49%); BVP: Bilateral Vestibular Paresis, 8 patients (10.43%); AUVL: 6 patients (7.43%); RD: Residual Dizziness after BPPV 10 patients (12.89%); VM:Vestibular Migraine 6 patients (7.46%); MD: Menière’s Disease: 6 patients (7.41%); Other: Myasthenia Gravis 1 patient, Acoustic Schwannoma, 2 patients (4.5%), Unknown 10 patients (12.5%).

Figure 5 resumes the diagnosis in the group of patients suffering from imbalance.

The diagnosis of BPPV, MD, VM, AUVL, PPPD was made in accordance with the international guidelines [7,8,9,10]. The diagnosis of vascular vertigo included patients affected by cerebellar stroke (one case in the group of the acute vertigo), Small Vessel Disease (18 in three groups), Vertebro-Basilar TIAs (11 in the three groups), orthostatic hypotension (one patient).

**Figure 6:**
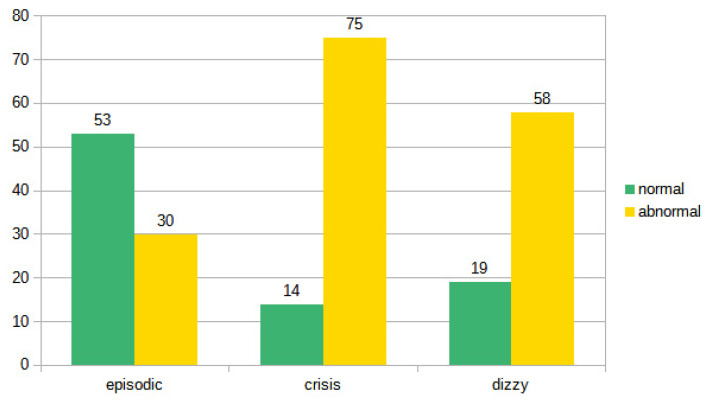
Distribution of normal and pathological examinations in the three subgroups.

On the basis of the results of the instrumental examination, we arbitrarily divided (for each type of symptoms) the patients in a group with a normal vestibular instrumental examination and a group of patients in which the clinical–instrumental evaluation showed some pathological results (i.e., canal paresis, spontaneous and/or positional nystagmus, altered vHIT, abnormal cVEMPs and/or oVEMPs). If we consider the results, the otoneurological examination (comparison between negative and non-negative diagnostic results), the difference between recurrent and acute vertigo and between recurrent vertigo and imbalance is highly significant (*p* < 0.01 in both cases). The difference between acute vertigo and imbalance was not significant (*p*: 0.157). The data seem to indicate a homogeneity between the group of vertigo examined during the acute phase and the patients observed because they were affected by ongoing imbalance. On the other hand, it seems that patients with recurrent vertigo more frequently exhibit a negative examination, and this is not surprising since they are often asymptomatic subjects examined in the intercritical phase (Figure 6).

A one-way ANOVA revealed that there was a statistically significant difference in episodic between crisis and imbalance (F = 29.97, df = 2, *p* = 0.000). Tukey’s HSD Test for multiple comparisons found that the mean value of episodic was significantly different between crisis and dizzy (*p* = 0.000, 95% C.I.). There was no statistically significant difference between crisis and dizzy (*p* = 0.375). The contingency tables in Bayesian statistics confirm the classic statistic: the BF _10_ independent multinomial of 4.663 × 10^8^ demonstrates the validity of the alternative hypothesis whereby the ‘episodic’ group is not equal to the ‘crisis’ group (‘Extreme’), and the Episodic group is not equal to the instability group (BF _10_ independent multinomial of 57,734.963, ‘Extreme’); however, the correspondence between the seizure group and that of instability is poor (BF _10_ independent multinomial of 0.428, ‘Anecdotal’).

## 4. Discussion

Ninety percent of episodic vertigo is related to one of the following vestibular diagnoses [1,6,11]:

(1) Benign paroxysmal positional vertigo.
(2) Vestibular Migraine.
(3) Menière’s disease.
(4) Vertebrobasilar TIAs.
(5) Orthostatic hypotension.

In addition, more rarely episodic vertigo could be due to other cause such as labyrinthine fistulas (of the lateral or superior canal), epilepsy, familial periodic ataxia type II, and vestibular paroxysmia. 

Our narrative review aims to simply indicate which are the most common vestibular pathologies that can produce the onset of recurrent episodes of vertigo, confirming that BPPV, VM and MD represent the most common causes. Altogether BPPV, VM, and MD seem to account for about 50% of patients presenting to the clinic for dizziness, as already reported [12]. Altogether, the three most common causes of vestibular vertigo have a 1-year prevalence of approximately 4.5% [13]. 

However, if on the one hand the diagnosis of BPPV can be quite easy on the basis of the anamnestic data and the results of the bedside examination, the diagnosis of MD and in particular of VM is more complex, given that in the initial stages of MD and in the intercritical phases of VM, the standard vestibular instrumental picture could be completely negative [14,15,16,17,18]. This implies that it is necessary to spend time collecting the detailed clinical history of the patient who sometimes is not able to exactly describe the symptoms with the risk of omitting important details for diagnostic purposes. For this reason, it is advisable to carefully evaluate some instrumental aspects that can be of valid help for a definitive diagnosis. However, the possibility of having an instrumental hallmark would be extremely useful, especially in cases where the clinical picture does not fully meet the international diagnostic criteria. We will try to consider the main alternative diagnoses from the point of view of an observer confronted with an asymptomatic patient, examined outside the crisis and who complains of relapsing episodic vertigo.

(1) BPPV from posterior and lateral canalithiasis. It is a vertigo typically evoked by head movements, which the patient generally remembers well and is able to report. Furthermore, the average duration of the symptoms is in the order of weeks [4], so the patient can be seen again in the acute phase: at that point we can also make the diagnosis of the previous episode. Horizontal canalolithiasis could have a shorter course [19,20] with more possibility to not be diagnosed, but the frequency of the recurrences and the characteristics of the vertigo are generally significant. It is practically impossible to find objectivity in the intercritical phase: only in some cases, ‘Ocular tilt reaction’ can be seen in the overcompensation stage but this does not allow a diagnosis of certainty [21,22]. A diagnosis in the event of a negative Dix–Hallpike maneuver is not acceptable: at most, a diagnostic doubt can be granted to be confirmed as soon as possible.
(2) According to the international guidelines, the diagnosis of VM is based exclusively on the clinical history and on the exclusion of other causes of vertigo [9]; therefore, it is always possible even in a non-critical phase (observing the migraine patient in the acute stage is rare and signs are not specific). In our series, VM is, as expected, a common cause of episodic vertigo; however, we found a large number of patients who presented with a first episode of acute vertigo who then developed a VM, confirming that VM pathology is, however, an increasingly recognized cause of acute vertigo [15]. We must keep in mind that following a first-ever presentation of vertigo (without a history of multiple episodes), it is often challenging to differentiate acute VM from other causes of acute vertigo, especially when acute VM is associated with positional vertigo and nystagmus thus mimicking BPPV [23]. Recently, the evaluation of a sign associated with migraine-associated vertigo has been proposed [24]: these patients, even outside the crisis, would show a pupillary nystagmus (“hippus”) characterized by alternating dilation and narrowing of the diameter of the pupils. This sign seems to be more easily (if not exclusively) detectable in patients suffering from migraine vertigo and would be linked to the alteration of the pupillary cycle of migraine as previously reported [25]. Therefore, it may be worthwhile to assess the presence or absence of pupillary nystagmus and further the medical history in case of inconsistency. Patients with VM frequently report a special sensitivity to head motion and complex visual surroundings [9]. Vestibular symptoms are generally triggered or worsened by self-motion and visual motion. These patients are prone to develop the so-called «Visual Dependence» (VD); this condition is defined as a reduced ability to disregard visual clues in complex or conflicting environments (supermarket, traffic) [26]. In brief, VD may correspond to the reduced ability to use complex visual input in a correct way in combination with an incorrect interaction with vestibular input. Since the migraine patient is very often a “visual dependent”, a negative or inconclusive clinical and instrumental examination associated with abnormal results of fHIT only under an optokinetic background could support the diagnosis of vestibular migraine [27]. These instrumental data could be helpful in correctly identifying patients with VM, particularly when the international diagnostic criteria are not completely met. Finally, in our experience, the effectiveness of a prophylactic therapy for VM (we reserved for cases with very frequent crises or in any case perceived as disabling) [11,28] could be useful as a diagnostic confirmation although some confounding factors (placebo effect, and spontaneous improvement) may interfere with the outcome of drug treatment [29].
(3) MD is a purely clinical diagnosis (except for audiogram): electrocochleography, VEMPs (cervical and ocular), video-HIT and even MRI with intratympanic gadolinium do not enter the diagnostic criteria for this disease [30]. However, in the early stages of the disorder, vestibular or cochlear symptoms may occur in isolation. Vertigo attacks are the most frequent symptom in the initial phase [31]. Less than 30% of MD patients have the three symptoms in the initial phase [32]. After 1 or 2 years, however, the complete set of symptoms is usually established. Patients with a longstanding history of recurrent isolated vertigo (e.g., no definitive auditory features) are therefore unlikely to have MD. The dissociation of thermal tests and video-HIT may be helpful [33,34], but the fundamental finding is the detection and documentation of fluctuating hearing loss.

Although MD and VM are considered among the main causes of recurrent episodic vertigo, in our series, there are patients affected by these pathologies who present symptoms characterized by chronic imbalance. This is not surprising since in the advanced phase of MD, it is extremely common to find sub-continuous postural instability accompanied by a clear reduction or disappearance of vertigo attacks. Similarly, some patients who fulfill all criteria for VM complain of the presence of sub continuous dizziness rather than vertigo, so much so that a recent consensus document from the Barany Society and the International Headache Society hopes that this symptomatology can in the future be considered in the classification of the VM [29].

(4) Vestibular TIAs in the posterior cranial fossa. Vertebrobasilar TIA is a common cause of isolated recurrent vertigo. The Vertebro-Basilar Insufficiency (VBI) can cause damage both at a central level, with involvement of the brainstem and cerebellum, and at a peripheral level, with involvement of the cochlea and vestibule, thus giving rise to an extremely polymorphic symptomatologic and objective picture. For this reason, circulatory insufficiency in the vertebro-basilar district is a common cause of vertigo, especially in subjects over 50, especially in the presence of vascular risk factors (smoking, diabetes, arterial hypertension and hyperlipemia); the high incidence of this symptom in the initial symptoms of VBI confirms the importance of posterior circulation disorders in the genesis of many vertigo syndromes [35]. The typical patient with vertigo caused by vertebro-basilar TIA is older than 60 years and has vascular risk factors such as history of smoking, hypertension, diabetes, or hyperlipidemia. Attacks may occasionally present with isolated vertigo but more commonly include associated symptoms from the posterior circulation territory. The duration of TIAs has been arbitrarily limited to 24 h; most, however, last minutes or 1 to 2 h. In this case, there are no residual signs in the intercritical phase and should be suspected in case of short-term, recurring vertigo [36]. They are often associated with other signs, always transient (visual disturbances, hearing loss, tinnitus, paresthesia, headache, etc.) but vertigo can be the only symptom. HINTs examination [37] is a valuable screening tool for distinguishing a central cause of acute vertigo (mainly cerebellar stroke) [38,39] from AUVL, such as vestibular neuritis, [40] but its value in case of VB-TIAs seems to be not so useful, due to the fact that after a transient ischemic attack no objective or instrumental signs are evident at the time of observation. On the contrary, the ABCD criterion (Table 1) remains valid, whose items can be reconstructed anamnestically, giving an idea of the circulatory defect in the posterior cranial fossa [41].

In addition to the presence of vascular risk factors), another condition needs to be considered: the presence of moderate or severe Small Vessel Disease, very common in older patients, can directly represent a cause of dizziness because of vascular injury to white matter tracts involved in postural balance or vestibular perception [42,43,44]. In our opinion, we believe that the concept of vascular vertigo should not be restricted to cases of stroke (resulting in acute vertigo with or without associated neurological signs) or TIA (resulting in symptoms characterized by recurrent episodes of vertigo). In our series, we have detected a significant number of subjects suffering from idiopathic chronic imbalance, in whom, alongside the presence of some vascular risk factors, the MRI study highlighted clear signs of SVD, the correlation of which with alterations of the cerebral microcirculation is well established as well as its association with “unexplained” dizziness [42]. Therefore, elderly patients with vascular risk factors who complain of impaired balance can be classified as vascular vertigo by virtue of the presence of microvascular injury especially in frontal white matter relevant to the control of balance in SVD [44].

On the contrary, the presence of recurrent vertigo episodes not clearly attributable to other causes must be taken into serious consideration (especially in patients with vascular risk factors) given that in 12–17% of the subjects affected by posterior circulation stroke, isolated recurrent vestibular symptoms were present in the 3 months before the ischemic attack [45,46].

(5) Orthostatic Hypotension (OH) is characterized by brief episodes of dizziness lasting seconds to minutes after standing up and can be diagnosed when a drop of systolic blood pressure of >20 mm Hg occurs after standing up [47]. Dizziness after standing up due to OH is a common type of dizziness with a lifetime prevalence of 12.5% and it may have severe consequences, causing syncope in 19% and traumatic injury in 5% of affected individuals [48]. Orthostatic hypotension can be associated with a “positional vertigo” (in reality it is a fainting sensation, but the patient often has difficulty describing it), in which the malaise does not manifest itself when taking the supine position but when assuming orthostatism or even prolonged standing. This symptom is typical of patients undergoing treatment with drugs that can cause hypotension or reduced alertness. In these cases, it is worthwhile to check the pressure and heart rate in orthostasis and in supine position.
(6) Perilymphatic fistula is an abnormal communication between the perilymphatic space and the outside, resulting in perilymph leakage [49]. Vertigo may be episodic, with attacks lasting seconds to days, or chronic, with superimposed fluctuations. Tinnitus and aural fullness may also occur. It can be a consequence of trauma or surgery, but the diagnostic problem arises in cases of idiopathic (spontaneous) fistula and may originate from straining or minor trauma on the background of a preexisting subclinical abnormality, such as local thinning of the labyrinthine capsule [49,50]. It may be enough to blow your nose, sneeze, laugh or even bend over to cause a fistula [51]. Audiological (sudden hearing loss, tinnitus, fullness in the ears) or vestibular (imbalance or vertigo) symptoms may be present, or some combination of them. It can enter into the differential diagnosis with vestibular migraine, endolymphatic hydrops, and functional vertigo. The problem is that there are no precise diagnostic criteria: the majority of the methods (audiovestibular examination, imaging, or laboratory testing for perilymph biomarkers) used to identify PF lacked the sensitivity and specificity to provide consistent diagnosis [49,51]. The diagnosis of PF is very challenging: a careful history (with the aim of evaluate a preceding event), associated with a complete battery of vestibular, auditory and high-resolution imaging studies (and, when possible, biomarker testing) is necessary before planning a surgical exploration of the middle ear [49,51]. In our experience, we encountered only one case of perilymphatic fistulae. We encountered a similar combination of auditory and vestibular symptoms in two patients suffering from Superior Canal Dehiscence Syndrome (SCDS) [52]. Perlymphatic fistula is an opening of the inner ear with a communication to the middle ear allowing fluid to move and stimulate the vestibular end organ in response to sound or pressure changes [53]. On the contrary, the symptomatology of SCDS is the consequence of a thinning or discontinuity of the bone that separates the superior canal from the middle cranial fossa [52]. The presence of this “third window” can disrupt the harmony of hearing and balance residing in the same otic capsule, leading to physiologic stimuli causing excitatory or ampullofugal deflection of the cupula of the superior semicircular canal. Patients with SCDS may experience symptoms of pressure- or sound-induced vertigo, hyperacusis to bone conducted sounds, and pulsatile tinnitus. Chronic disequilibrium is common. The diagnosis is based on the presence of one of the previous described symptoms in association with a high resolution CT scan reformatted in the plane of the superior SC demonstrating a dehiscence and at least one of the following diagnostic test: negative bone conduction on pure tone audiometry, low cervical VEMPs threshold or high ocular VEMP amplitude, and elevated summating potential to action potential ratio on electrocochleography in the absence of a sensorineural hearing loss [54,55].

Evaluating the aggregate results of our retrospective evaluation of three groups of patients with different types of presentation of their symptomatology, comparing only the patient with negative vestibular examination from those in which a definite diagnosis was made, we found a highly significant difference between recurrent and acute vertigo and between recurrent vertigo and imbalance (*p* < 0.01 in both cases). On the contrary, the difference between acute vertigo and imbalance was not significant (*p*: 0.157). The data seem to indicate a homogeneity between the group of vertigo examined during the acute phase and the patients observed because they were affected by ongoing imbalance. On the other hand, it seems that patients with recurrent vertigo more frequently exhibit a negative examination, and this is not surprising since they are often asymptomatic subjects examined in the intercritical phase. 

Of course, when the clinical (bedside) and instrumental examination do not allow to drive a definite diagnosis, a precise and in-depth research of the patient’s clinical history, inducing him to provide details that may escape the patient himself, could be an important the key to suspect or make a diagnosis. For example, in the diagnosis of vestibular migraine, the presence of accompanying symptoms (phono-photophobia and visual aura, motion sickness, besides the presence of migraine) is a fundamental element to be considered. 

Similar considerations can be made regarding MD. Probably, some cases of episodic vertigo that we considered as due to “unknown cause” could be included in the so-called “Benign Recurrent Vertigo, BRV”. BRV, better defined as recurrent vestibular symptoms non-otherwise-specified (RVS-NOS), is characterized by recurrent spontaneous and/or positional vertigo (spinning or non-spinning) attacks without hearing loss, tinnitus, aural fullness and with no associated non-vestibular migrainous symptoms [56,57]. These patients do not meet the criteria for VM, BPPV or MD and, for this reason, this syndrome cannot be described as a separate clinical entity. Van der Leeuwen et al. [57] studied a group of patients with BRV followed-up for more than 3 years, showing a progressive disappearance of vertigo attacks leading to the consideration of BRV as a mild or incomplete variant of VM and MD, rather than a separate disease entity with distinct pathognomonic features. On the contrary, Dlugaiczyk et al. [56] studied and followed up for more than 5 years a group of patients with RVS-NOS, indicating no female preponderance (opposite to VM), milder associated neurovegetative symptoms, and a clinical presentation more similar to VM than MD. The stability of symptoms over time with a low rate of conversion into another diagnosis (VM or MD) lead the authors to consider RVS-NOS as a vestibular entity composed of several disorders, including a spectrum of mild or incomplete variants of well-known vestibular disorders, rather than a separate clinical entity [57]. Recently, on the basis of a similar temporal profile and the presence of migraine precursors (motion sickness in pediatric age) between RVS-NOS and VM, a common pathophysiological mechanism could be hypothesized [58]. 

Finally, we must not forget the important economic impact of episodic vertigo, the presence of which involves not only a high number of visits to outpatient and/or emergency departments, but also the frequent use of imaging, whose usefulness appears limited to excluding infrequent pathologies of the central nervous system or less common pathologies as SSCDS, but rather to reassure the patient about the not life-threatening nature of the complained symptomatology. This is particularly true considering that the three major causes of episodic vertigo (BPPV, VM, and MD) are purely clinical diagnoses for which current guidelines do not provide for the use of radiological or paraclinical criteria. In this regard, even the use of a simple test such as SVINT could help differentiate MD from VM. It has been shown that in the interictal period of these two diseases, the positivity rate is much higher (60%) in MD than in VM (6%) [59]. Considering that the SVIN is a typical hallmark of partial or total UVL, it is likely to believe that the different positivity rate is attributable to different pathophysiological mechanisms, e.g., peripheral in MD and likely related to central vestibular involvement in VM [59].

Therefore, an accurate collection of anamnestic data and a careful bedside examination, perhaps accompanied in doubtful cases by a targeted instrumental evaluation, represent fundamental elements for quickly reaching a correct diagnosis with a consequent reduction in direct and indirect health costs, such as lost productivity, occupational absenteeism and disability, stress, and anxiety [60]. 

## 5. Conclusions

Episodic vertigo may be a diagnostic problem because the patient is often examined at a stage when he or she is asymptomatic; consequently, it often happens that the vestibular examination is negative. An anamnesis aimed at confirming or excluding the main causes of recurrent vertigo and the search for specific signs can allow diagnosis in most cases, even when the duration of the vertigo is too short to allow the patient to return to the acute phase. The main diseases to look for are BPPV, VM, vascular vertigo and Orthostatic Hypotension, which alone are almost all the pathologies that cause episodic vertigo. An highly indicative clinical–anamnestic element is represented by the search for information on the duration of the recurrent attacks of vertigo: recurrent rotatory vertigo attacks lasting a few seconds are typical of BPPV (in which there is the fundamental distinctive element represented by the typically positional character of vertigo—the reason why this disease is excluded in the lecture), but this feature could be present in Perilymphatic fistula, Vertebro-Basilar TIA and, more rarely, in the so-called “Vestibular Paroxysmia”; however, it was never diagnosed in our series. Surely, a longer duration is typical of patients suffering from MD and/or VM. However, in case of episodic vertigo, an accurate and complete instrumental evaluation can be extremely useful in cases where the anamnestic picture reported by the patient is not entirely compatible with the diagnostic criteria, especially when a diagnosis of VM or MD is suspected.

## Figures and Tables

**Table 1:** ABCD score. A score of 3 or greater is suspicious for a TIA predictive of stroke. BP: Blood Pressure.

Clinical Factor	Score
Age 60 years or older	1
BP ≥ 140/90 mm Hg	1
Clinical: speech disturbance	1
Clinical: unilateral weakness	2
Duration < 10 min	0
Duration 10–59 min	1
Duration 10–59 min	1

## Data Availability

Not applicable.
